# Impact of Carbohydrate Counting Method during Pregnancy in Women with Pregestational Diabetes Mellitus: A Controlled Clinical Trial

**DOI:** 10.1055/s-0042-1742291

**Published:** 2022-02-09

**Authors:** Larissa Mello de Oliveira, Gabriella Pinto Belfort, Patricia de Carvalho Padilha, Eliane Lopes Rosado, Letícia Barbosa Gabriel da Silva, Sanmira Fagherazzi, Lenita Zajdenverg, Roberto Luís Zagury, Claudia Saunders

**Affiliations:** 1 Programa de Pós-graduação em Nutrição do Instituto de Nutrição Josué de Castro da Universidade Federal do Rio de Janeiro (UFRJ), Rio de Janeiro - RJ, Brazil; 2 Maternidade Escola da UFRJ, Rio de Janeiro – RJ, Brazil

**Keywords:** pregnancy, diabetes mellitus, prenatal care, nutrition therapy, gravidez, diabetes mellitus, cuidado pré-natal, terapia nutricional

## Abstract

**Objective** To evaluate the effect of the carbohydrate counting method (CCM) on glycemic control, maternal, and perinatal outcomes of pregnant women with pregestational diabetes mellitus (DM).

**Methods** Nonrandomized controlled clinical trial performed with 89 pregnant women who had pregestational DM and received prenatal care in a public hospital in Rio de Janeiro, state of Rio de Janeiro, Brazil, between 2009 and 2014, subdivided into historic control group and intervention group, not simultaneous. The intervention group ( *n* = 51) received nutritional guidance from the carbohydrate counting method (CCM), and the historical control group ( *n* = 38), was guided by the traditional method (TM). The Mann-Whitney test or the Wilcoxon test were used to compare intra- and intergroup outcomes and analysis of variance (ANOVA) for repeated measures, corrected by the Bonferroni post-hoc test, was used to assess postprandial blood glucose.

**Results** Only the CCM group showed a reduction in fasting blood glucose. Postprandial blood glucose decreased in the 2 ^nd^ ( *p* = 0.00) and 3 ^rd^ ( *p* = 0.00) gestational trimester in the CCM group, while in the TM group the reduction occurred only in the 2 ^nd^ trimester ( *p* = 0.015). For perinatal outcomes and hypertensive disorders of pregnancy, there were no differences between groups. Cesarean delivery was performed in 82% of the pregnant women and was associated with hypertensive disorders (gestational hypertension or pre-eclampsia; *p* = 0.047).

**Conclusion** Both methods of nutritional guidance contributed to the reduction of postprandial glycemia of women and no differences were observed for maternal and perinatal outcomes. However, CCM had a better effect on postprandial glycemia and only this method contributed to reducing fasting blood glucose throughout the intervention.

**ReBEC Clinical Trials Database** The present study was registered in the ReBEC Clinical Trials Database (Registro Brasileiro de Ensaios Clínicos, number RBR-524z9n).

## Resumo

**Objetivo** Avaliar o efeito do método de contagem de carboidratos no controle glicêmico, desfechos maternos e perinatais de gestantes com diabetes mellitus (DM) pré-gestacional.

**Métodos** Ensaio clínico controlado não randomizado realizado com 89 gestantes com DM pré-gestacional atendidas em hospital público do Rio de Janeiro, RJ, Brasil, entre 2009 e 2014, divididas em grupo controle histórico e grupo intervenção. O grupo intervenção ( *n* = 51) recebeu orientação nutricional com base no método de contagem de carboidratos (CCM) e o grupo controle histórico ( *n* = 38) foi orientado pelo método tradicional (MT). Os testes de Mann-Whitney ou de Wilcoxon foram usados para comparar os desfechos intra- e intergrupos e, para avaliar a glicemia pós-prandial, análise de variância (ANOVA, na sigla em inglês) para medidas repetidas foi usada.

**Resultados** Somente o grupo com método CCM apresentou redução da glicemia de jejum. A glicemia pós-prandial diminuiu no 2° ( *p* = 0,00) e 3° ( *p* = 0,00) trimestres gestacionais no grupo com método CCM, e no grupo com método tradicional, a redução ocorreu apenas no 2° trimestre ( *p* = 0,015). Para os resultados perinatais e distúrbios hipertensivos da gravidez, não houve diferenças entre os grupos. O parto cirúrgico foi realizado em 82% das gestantes e esteve associado a distúrbios hipertensivos gestacionais ( *p* = 0,047).

**Conclusão** Ambos os métodos de orientação nutricional contribuíram para a redução da glicemia pós-prandial e não foram observadas diferenças para os resultados maternos e perinatais. No entanto, o método CCM apresentou melhor efeito sobre a glicemia pós-prandial e foi o único que induziu redução da glicemia de jejum.

## Introduction

Diabetes mellitus (DM) is a major public health problem. It is estimated that 15.8% of pregnant women present with hyperglycemia during pregnancy, that the proportion of cases of DM detected before pregnancy is of 7.9%, and that the prevalence of gestational DM in Brazil is of 18%. 1 2 This scenario requires specialized care and adequate management to ensure better obstetric and perinatal outcomes. 3

Nutritional guidance is an important component of the treatment of individuals with DM, especially during pregnancy. To achieve the desired objectives, the eating plan must be given individually, by trained professionals acting together with a multidisciplinary team, and with the aim of promoting glycemic control and adequacy of maternal weight gain, thereby minimizing adverse outcomes. 3 The nutritional orientation based on distributing daily energy intake across meals, referred to here as the traditional method (TM), and the carbohydrate counting method (CCM) are both dietary guidance strategies used for individuals with DM. 3

In CCM, the idea is to control the total amount of carbohydrates, in grams, consumed in each meal based on the fact that the quantity is more important than the type or source of carbohydrates, because they will all be transformed into glucose. 4 5 Based on a study conducted by The Diabetes Control and Complications Trial, CCM has emerged as an innovative alternative as it provides greater dietary flexibility and better glycemic control in individuals with type 1 DM (T1DM). 6

The beneficial effect of CCM in reducing glycated hemoglobin in adults with DM1 has also been described by Bell et al. 7 in a systematic review of clinical trials. Gabriel da Silva et al. 8 found that TM and CCM can be used in the nutritional orientation of pregnant women with gestational DM without differences for glycemic control. The American Diabetes Association recommends that people with DM1 and type 2 DM (T2DM), especially those on insulin therapy, adopt CCM to improve glycemic control. 3

The present study evaluates the comparative effects of TM and CCM as dietary guidance methods on glycemic control and maternal and perinatal outcomes in pregnant women with DM.

## Methods

### Study Design

The present nonrandomized, controlled, clinical trial was conducted at the maternity teaching hospital (Maternidade Escola) of the Universidade Federal do Rio de Janeiro, Rio de Janeiro, state of Rio de Janeiro, Brazil, which is a reference for the care of pregnant women with DM who are residents of the state of Rio de Janeiro, Brazil.

### Population and Sample Size

**Fig. 1:**
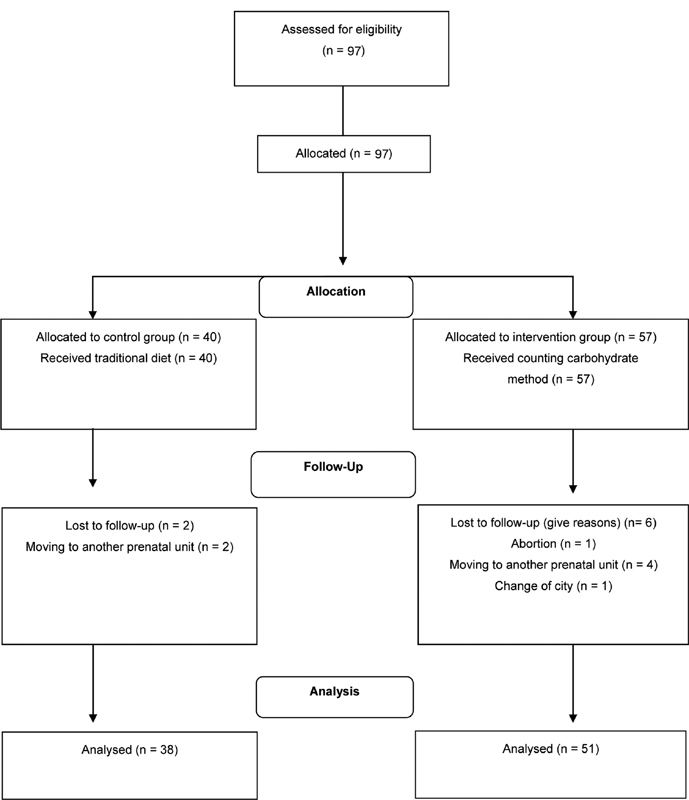
Summary of patient flow (2009–2014).

The study population consisted of adult pregnant women diagnosed with T1DM or T2DM and others, with diagnosis confirmed by the doctor, who received antenatal and postpartum care from a multidisciplinary team at a the Maternidade Escola of the Universidade Federal do Rio de Janeiro, between 2009 and 2011 and between 2011 and 2014. The inclusion criteria were: age ≥ 20 years old, single pregnancy, gestational age at the first care visit with a nutritionist < 28 weeks, and attendance of antenatal care visits at the maternity hospital. Women with diabetic nephropathy, syphilis under treatment, and HIV infection were not eligible. The initial study sample comprised 97 women, 40 of whom were put in the control group and 57 in the intervention group. Figure 1 presents the flowchart of the final sample ( *n* = 89; from 2009 to 2014).

### Groups

**Traditional group (TM group):** was the historical control group. To construct the group, the medical records of all women who attended the maternity hospital from June, 2009 to June, 2011 were consulted. All women who met the inclusion criteria were included. All members of the group received antenatal nutritional care and routine nutritional intervention based on TM without the intervention of a researcher.

**Intervention group (CCM group):** consisting of pregnant women with pregestational DM who received antenatal care between July, 2011 and October, 2014. All women who met the inclusion criteria were included in the study and received the antenatal nutritional intervention based on CCM.

The data were collected from the medical records of both groups and also from interviews with the intervention group (CCM). The groups were not followed simultaneously.

### Nutritional Intervention

The women from the control group were guided by TM and received dietary guidelines with a list of food replacements composed of nine groups (fruits, breads, dairy products, meats, cereals, legumes, fats, and vegetables). The foods and preparations were presented in portion sizes according to their energy value. The women were discouraged from consuming sucrose. 3

The women from the intervention group were guided by CCM and received an individualized dietary form with a list of food substitutions grouped in portions containing 15 g of carbohydrates and classified into food groups. 9 They were also given recipes for special occasions, which also contained a carbohydrate count. The use of sucrose was discouraged and limited to 10% of the total energy intake (TEI). 10

Both groups received their diet printed on colored paper with figures representing the suggested foods for each meal. The dietary plan was designed to reach the recommended gestational weight gain and was adapted to the eating habits and sociodemographic conditions of the women, based on the precepts of healthy eating. 11 The dietary plan was adjusted at each care visit according to any digestive symptoms reported, maternal comorbidities, adherence, glycemic control, and weekly weight gain. The women from both groups were instructed on the consumption of sweeteners. 9

The proportion of macronutrients was equal for both groups, with carbohydrates accounting for between 50 and 52% of the TEI, proteins accounting for between 18 and 20% of the TEI, and fats accounting for between 30 and 33% of the TEI, including < 7% saturated fats. 12 13 Micronutrient recommendations were also met. 12

The dietary plan was divided into five to six meals a day, with regular schedules for both groups. The following energy distribution was adopted for both groups: breakfast and afternoon snack: between 10 and 15% of the TEI; small morning and evening snacks: between 5 and 10% of the TEI; and lunch and dinner: between 20 and 30% of the TEI. 3

All women received specific guidelines for management in cases of hypoglycemia (capillary glycemia < 65mg/dl) and hyperglycemia (fasting glucose level > 95mg/dl and/or glucose level > 140mg/dl 1 hour after a meal). 3 5

Insulin doses were stipulated by the endocrinologist based on the gestational weight of the patient. In addition to dietary guidance, all pregnant women using insulin performed self-monitoring of blood glucose (SMBG), at least 4 times a day, prioritizing the measurement 1 hour after the 3 main meals and fasting.

Pregnant women in the CCM group were initially instructed to keep the insulin/carbohydrate ratio at 1:15. Both groups were instructed to correct using prandial insulin (regular or fast analog) according to the table, whose intervals were determined by calculating the sensitivity factor in each consultation. Until the 29 ^th^ week, the dose adjustment was made by the specialist doctor every fortnight, and from the 30 ^th^ week, weekly. Glycemic targets were preprandial > 65 and < 95 mg/dl and 1-hour postprandial < 140 mg/dl. In the pregnant women with unannounced hypoglycemia, the limits could be adjusted to avoid the risk of severe hypoglycemic events.

### Nutritional Assessments

#### Anthropometric Assessments

Nutritional assessments were done for both groups, and the anthropometric assessment covered pregestational weight (kg), reported or measured up to the 13 ^th^ gestational week using a Balmak electronic scale, and height (m) measured in the first appointment by a stadiometer attached to the scale. The classification of pregestational body mass index (BMI), weekly and total recommended weight gain was analyzed and estimated for each woman and then recalculated at each visit, according to recommendations of the Institute of Medicine. 13

#### Biochemical and Clinical Assessment

Gestational hypertension was set at arterial pressure ≥ 140 × 90 mmHg diagnosed after the 20 ^th^ gestational week; pre-eclampsia-hypertension and proteinuria was set at ≥ 300 mg/24 hours; and eclampsia was onset of seizures associated with hypertension. 14 Fasting blood glucose was measure after 8 hours of fasting and 1 hour postprandial blood glucose measurement was part of the prenatal examination routine and was measured in the 1 ^st^ , 2 ^nd^ and 3 ^rd^ trimesters of pregnancy, using the enzymatic colorimetric method. Good glycemic control was set at fasting glucose ≤ 95 mg/dL and postprandial glucose (1 hour) ≤ 140 mg/dL. 5

#### Sociodemographic, Biological and Obstetric Assessment

The participants were considered as having adequate sanitation when their dwellings had regular garbage collection, piped water, and sewage collection. When any one of these services was absent, sanitation was considered as inadequate. The following characteristics were evaluated: maternal age at delivery; skin color, educational level; marital status; type of diabetes, time of illness; presence of comorbidities, and occupation. The number of appointments for prenatal care and nutritional prenatal care were identified.

### Adherence to Dietary Guidance

Adherence to the diets was by an instrument that evaluated four criteria related to dietary pattern and weekly gestational weight gain. 15 Adherence was then classified as poor, when the woman did not meet any criteria or met only one; good adherence when women met two or three criteria, and great adherence when women met four criteria.

### Outcomes

The outcomes studied were: glycemic control per trimester (fasting blood glucose – mg/dL and post prandial glucose – mg/dL); gestational weight gain (kg); presence of any hypertensive disorder of pregnancy (yes/no); pre-eclampsia (yes/no); type of delivery (vaginal/caesarean); gestational age at birth (weeks); preterm birth (< 37 weeks/ ≥ 37 weeks); Apgar score in the 1 ^st^ and 5 ^th^ minutes; cephalic perimeter and length (cm); macrosomia (yes/no); low birthweight (yes/no). Low birthweight was set at < 2,500 g and macrosomia at ≥ 4,000 g. 16 The exposure variable was the study group (TM group or CCM group).

### Data and Statistical Analysis

For the characterization of the sample, measures of central tendency and sample dispersion (mean and standard deviation [SD] or median and interquartile range [IQR]) and relative and absolute proportions were used. Continuous variables were analyzed using the Student *t* -test or the Mann-Whitney test, according to the normality of the data. The Chi-squared test was used to compare proportions between groups. The normality of variables was tested by the Shapiro-Wilk test. To compare fasting blood glucose between groups, the Mann-Whitney U test was used, and for the intragroup comparison, the Wilcoxon test was used. To compare postprandial glucose between groups, analysis of variance (ANOVA) was used for repeated measures; maternal age was included in the model as covariate and was corrected by the Bonferroni post-hoc test. The significance level considered was a p-value < 0.05 and 95% confidence intervals (CIs) or IQRs were estimated, when necessary. The analyses were performed using IBM SPSS Statistics for Windows, version 22.0 (IBM Corp., Armonk, NY, USA).

### Sample Size

A convenience sample was used, considering the small number of cases of pregnant women with DM receiving care at the maternity hospital. Given that ∼ 40 women with DM receive care at the hospital every year, a sample size of 40 was estimated for each group.

## Results

A total of 89 women were studied ( Fig. 1 ). The sociodemographic and anthropometric characteristics of the women were found to be similar in both groups, as were the maternal and antenatal care characteristics, except for maternal age, which was higher in the CCM group ( Table 1 ). However, maternal age was not correlated with fasting and postprandial blood glucose levels neither in the 1 ^st^ trimester ( *p* = 0.10; *p* = 0.55), the 2 ^nd^ trimester ( *p* = 0.09; *p* = 0.74), or the 3 ^rd^ trimester ( *p* = 0.06; *p* = 0.22), respectively. As for the type of DM, 47% ( *n* = 18) of the women had T1DM in the TM group and 33% ( *n* = 17) in the CCM group. Six women had diabetes without classification, three had Maturity Onset Diabetes of Young (MODY) diabetes, and one had Latent Autoimmune Diabetes of the Adult (LADA) diabetes, but there was a similarity between the groups, according to the type of DM ( Table 1 ). The chronic diseases identified were hypothyroidism (TM *n* = 3; CCM *n* = 3) and chronic hypertension (TM; *n* = 3, CCM *n* = 10).

**Table 1:** Anthropometric and sociodemographic characteristics of pregnancy according to study groups. Rio de Janeiro, RJ, Brazil (2009-2014)

Maternal characteristics	TM group	CCM group	*p-value*
**Diabetes, type (n, %)**			0.12
Type 1	18 (47.4)	17 (33.3)	
Type 2	15 (39.5)	29 (56.9)	
Others *	5 (13.1)	5 (9.8)	
**Occupation (n,%)**			
Works	19 (50)	32 (61.7)	0.28 a
Does not work	19 (50)	19 (38.3)	
**Marital status (n, %)**			
Stable union / married	28 (73.7)	39 (76.5)	0.81 a
Single, divorced or widowed	10 (26.3)	12 (23.5)	
**Level of education (n,%)**			
Incomplete high school	8 (34.8)	17 (34.0)	1.00 a
Complete high school	15 (65.2)	33 (66.0)	
**Housing sanitation** conditions (n,%) **			
Inadequate	8 (21.1)	4 (7.8)	0.12 a
Adequate	30 (78.9)	47 (92.2)	
**Skin color (n, %)**			
Brown or black	7 (43.8)	29 (61.7)	0.25 a
White	9 (56.2)	18 (38.3)	
**Maternal age at delivery, years old**			0.01 b
Median (IQR)	28.47 (24.00–32.00)	32.07 (27.00–37.00)	
N	36	51	
** Pregestational BMI, kg/m ^2^ . Median (IQR) **	25.69 (21.34–28.69)	27.77 (23.50–32.67)	0.10 b
N	37	51	
**Diagnostic time of diabetes, years** Median (IQR) N	8.20 (3.00–13.00) 37	8.30 (2.00–12.25) 50	0.89 b
**Gestational age at the first prenatal visit, weeks** Median (IQR) N	12.21 (9.00–13.25) 38	12.60 (9.00–16.00) 51	0.73 b
**Number of prenatal care** Consultations. Median (IQR) N	12.84 (11.00–15.25) 38	12.61 (9.00–16.00) 51	0.25 b
**Number of consultations with the nutritionist**			0.13 b
Median (IQR) N	5.66 (3.00–7.25) 38	6.39 (5.00–7.00) 51	

Abbreviations: BMI, Body Mass Index; CCM, Counting Carbohydrate method group; IQR, Interquartile range; n, sample number; TM, Traditional method group.

a p-value to compare proportions was obtained by the Chi-squared test.

b p-value for median was obtained by the Mann-Whitney test.

* others: Diabetes MODY, LADA or without classification.

** adequate housing with regular garbage collection, treatment of sewage and piped water, inadequate - when one of the services was absent.

The fasting blood glucose was lower in the TM group in the 1 ^st^ trimester (median = 95 mg/dl and 135 mg/dl for the CCM group, *p* = 0.01) and no significant changes for fasting blood glucose were observed in the TM group across the intervention. However, the CCM group showed reduction of fasting glucose in all gestational trimesters ( Table 2 ).

**Table 2:** Evolution of fasting blood glucose in pregnant women with previous diabetes. Rio de Janeiro, RJ, Brazil (2009-2014)

Outcomes	TM group				CCM group				
	n	Median	IQR	p a	n	Median	IQR	p a	p b
Fasting blood glucose, 1 ^st^ trimester (mg/dL)	25	95.0	81.5-129.5	-	39	135.0	113.0–160.0	-	0.01
Fasting blood glucose, 2 ^nd^ trimester (mg/dL)	37	104.0	78.8–140.5	0.14	48	110.5	91.1–141.5	0.01	
Fasting blood glucose, 3 ^rd^ trimester (mg/dL)	37	90.0	79.0–122.0	0.20	46	91.6	82.1–114.8	0.00	
Mean change of fasting blood glucose during study (mg/dL) *	24		22.1 ± 58.7		35		39.01 ± 41.9		0.20

Abbreviations: CCM, Counting Carbohydrate method group; n, sample number; TM, Traditional method group.

a p-value obtained by the Wilcoxon test for the intragroup evaluation.

b p-value obtained by the Mann Whitney test for the between groups evaluation.

* Results expressed in mean change and standard deviation of fasting blood glucose between the 1 ^st^ and 3 ^rd^ gestational trimesters.

**Fig. 2:**
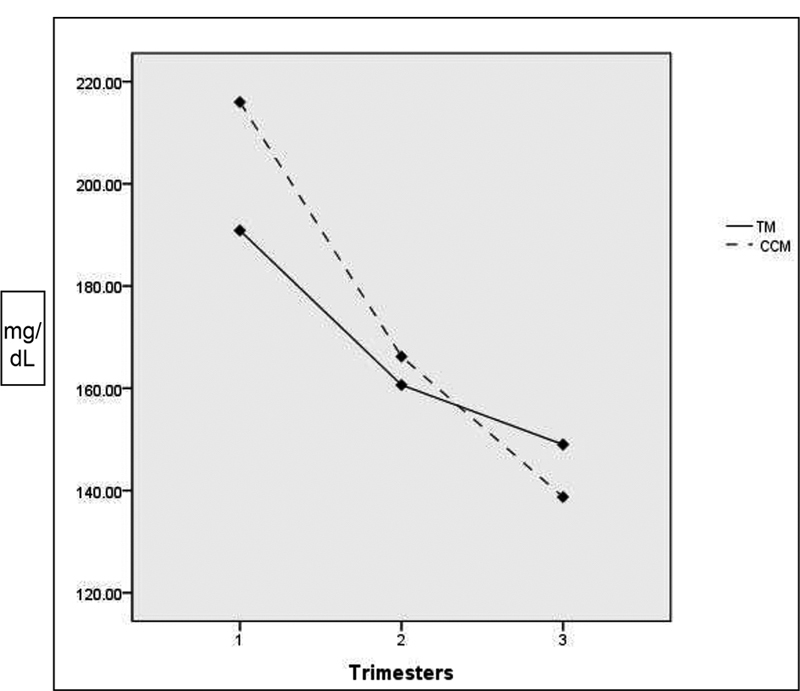
Evolution of the postprandial glucose in the intervention. ANOVA for repeated measures with maternal age adjusted and Bonferroni correction. **Traditional method group** : 1 ^st^ to 2 ^nd^ trimester ( *p* = 0.015), 2 ^nd^ to 3 ^rd^ trimester ( *p* = 0.798), and between the 1 ^st^ and 3 ^rd^ trimesters ( *p* = 0.034). For **Carbohydrate counting method group** : 1 ^sd^ to 2 ^nd^ trimester ( *p* = 0.000), 2 ^nd^ to 3 ^rd^ trimester ( *p* = 0.009), and between the 1 ^st^ and 3 ^rd^ trimesters ( *p* = 0.000).

For the postprandial glucose, there was no difference between groups ( *p* = 0.539), but both groups showed a reduction in postprandial blood glucose throughout the intervention. Nevertheless, the CCM group showed reduction in all gestational trimesters, while the TM group showed no reduction between the 2 ^nd^ and 3 ^rd^ gestational trimesters ( Fig. 2 ). Regarding gestational weight gain, no differences were observed between groups ( *p* = 0.147 ( Table 3 ).

**Table 3:** Newborn and maternal characteristics, according to the study groups. Rio de Janeiro, RJ, Brazil (2009-2014)

Characteristics	TM group	CCM group	*p* - *value*
Birthweight, grams (mean, SD)	3193.8 (648.30)	3248.1 (641.50)	0.69 a
*n*	38	48	
Gestational age at birth, weeks (median, IQR)	38.00 (37.00- 39.00)	38.00 (36.00–38.00)	0.61 b
*n*	38	48	
Cephalic perimeter, cm (median, IQR)	34.50 (33.00–36.00)	34.00 (33.00–36.00)	0.97 b
*n*	38	42	
Length, cm (mean, SD)	48.04 (3.18)	48.08 (3.14)	0.95 a
*n*	38	42	
Apgar, 1 ^st^ minute (median, IQR)	8.00 (8.00–9.00)	8.00 (7.25–9.00)	0.74 b
*n*	38	48	
Apgar, 5 ^th^ minute (median, IQR)	9.00 (9.00–9.00)	9.00 (9.00–9.00)	0.64 b
*n*	38	48	
Gestational weight gain, kg (median, IQR)	13.34 (9.53–17.47)	11.00 (8.90–15.35)	0.14 b
*n*	36	49	
Hypertensive disorders of pregnancy (n, %)			
Yes	11 (28.9)	23 (45.1)	0.12 c
No	27 (71.1)	28 (54.9)	
Pre-eclampsia (n, %)			
Yes	7 (18.4)	13 (25.5)	0.43 c
No	31 (81.6)	38 (74.5)	
Type of delivery (n, %)			
Vaginal	9 (23.7)	5 (10.2)	0.09 c
Cesarean	29 (76.3)	44 (89.8)	
Macrosomia (n, %)			
Yes	5 (13.2)	4 (8.3)	0.50 c
No	33 (86.8)	44 (91.7)	

Abbreviations: cm, centimeters; n, sample number; CCM, carbohydrate method; IQR, Interquartile range; p, p-value; SD, standard deviation; TM, traditional method.

a Student *t* -test.

b Mann-Whitney test

c Chi-squared test.

Among the outcomes studied, the total prevalences of macrosomia, low birthweight, and prematurity were 10.5% ( *n* = 9), 12.8% ( *n* = 11), and 38.4% ( *n* = 33), respectively, with no differences between groups. The type of delivery and other characteristics of the newborn were also similar between groups. Hypertensive disorders of pregnancy affected 38.2% of the women and 22.5% ( *n* = 20) were cases of pre-eclampsia, but there was no case of eclampsia and there was no difference between groups ( Table 3 ). Cesarean delivery was performed in 82% of the participants and was associated with the presence of hypertensive disorders of pregnancy ( *p* = 0.047–data not shown in tables).

On comparing the adherence levels between the groups, no difference was observed for the 2 ^nd^ , 3 ^rd^ , 4 ^th^ , 5 ^th^ or 6 ^th^ visits ( *p* = 0.13; *p* = 0.83; *p* = 0.26; *p* = 0.10; *p* = 1.00, respectively).The adherence increase with the intervention time; in the 2 ^nd^ visit, 29.6% in the TM group and 47.8% in the CCM group had a good or great adherence; in the 4 ^th^ visit, 47.6% in the TM group and 62.2% in the CCM group had a good or great adherence; and in the 6 ^th^ visit, 83.3 and 84% of pregnant women in he TM group and in the CCM group, respectively, had a good or great adherence (data not shown in tables).

## Discussion

The results of the present study suggest that there was no association between dietary guidance and maternal and perinatal outcomes, suggesting that both nutritional approaches could be adopted in the clinical practice with pregnant women with DM. 17 Pregnant women in both groups had an important reduction in postprandial glucose. Moreover, the CCM group showed a better control of fasting blood glucose and of postprandial glucose in the 3 ^rd^ trimester.

Perichart-Perera et al., 17 in a study of pregnant women with T2DM, described that the adoption of traditional nutritional guidance method was beneficial, since it was associated with a lower risk of pre-eclampsia, hospitalization, and neonatal death than the control group, which did not receive individualized nutritional treatment.

The benefits of the traditional nutritional guidance method have been demonstrated for a long time. 3 18 However, Christensen et al. 18 suggest that while this method may be useful for many pregnant women, it is rigorous and allows for little flexibility. Huang et al. 19 found that individualized nutritional guidance prescribed by a nutritionist based on CCM improves glycemic control in patients with T2DM. However, this study did not compare the use of CCM with an individualized dietary method, and the control group received only general guidelines in group settings with a nurse. It is noteworthy that the cited studies were performed with patients with T1DM or T2DM, and to our knowledge there are no studies that have evaluated the effect of these methods on pregnant women with DM.

The observed similarity between baseline groups is important for clinical trials. Although differences were observed between the groups in relation to fasting blood glucose in the 1 ^st^ trimester, it should be noted that the tests were performed before the 1 ^st^ care visit with the nutritionist and were not influenced by the form of dietary guidance. This difference can be explained in part by the higher maternal age of the CCM group.

The present study showed that glycemic control improved during gestation. This improvement in glycemic control may have been due to improved adherence to prenatal nutritional care associated with an increased number of care visits with a nutritionist, as well as to the work of the multidisciplinary team specialized in the treatment of pregnant women with DM. The improvement in adherence to dietary guidance may have favored the formation of a bond between the professional and the women. The strategies to improve adherence and bonding were the fact that the women saw the same nutritionist throughout their antenatal care and received individualized guidance. 15

Although the participants in the CCM group were older, only this group experienced a reduction in fasting blood glucose and a reduction in postprandial blood glucose between the 2nd and 3rd gestational trimesters. This suggests that the CCM allows better control over blood glucose, since older age is associated with a greater chance of high blood glucose. 20 21

The CCM has been associated with lower serum concentrations of glycated hemoglobin in pregnancy and in pregnant women with T1DM, which probably occurred due to the fact that this method allows greater control over the amount of carbohydrate consumed, which influences the serum concentrations of postprandial glucose. 22

While the CCM group had better control over postprandial blood glucose between the 2 ^nd^ and 3 ^rd^ trimesters of pregnancy, this cannot be reflected in the proportion of macrosomia, due to the fact that study participants initiate pregnancy with an uncontrolled glycemic level. Bashir et al. 23 identified that glycated hemoglobin in the 1 ^st^ and 3 ^rd^ trimester, which is related to higher glycemic levels, was associated with a greater chance of macrosomia in pregnant women with T2DM.

Another strong point for the CCM is that, despite having the highest average maternal age, there was no difference of macrosomia prevalence between groups. Dai et al., 24 in a systematic review of the literature with meta-analysis, identified that advanced maternal age (between 35 and 39 years old), regardless of the presence of diabetes or of other confounding factors, increases the risk of macrosomia (odds ratio [OR] = 1.42; 95%CI: 1.25–1.60).

Pregnant women with DM have a higher risk of macrosomia. The prevalence of macrosomia described in a study by Manderson et al. 25 of pregnant women with T1DM was of 21%, and Owens et al., 26 who evaluated maternal and neonatal outcomes in Irish pregnant women with T1DM and T2DM, observed a prevalence of macrosomia of 30 and 20%, respectively. 25 26 The low prevalence of macrosomia described in our study can be attributed to the quality of the antenatal care given by the hospital performed by a multidisciplinary team specialized in the care of women with DM.

No difference was observed between groups for perinatal outcomes, but it is known that episodes of hyperglycemia during pregnancy with gestational DM, not frequently treated, were associated in the HAPO study, with the presence of glucose intolerance in their children at between 10 and 14 years old, independent of maternal and childhood BMI and family history of diabetes. 27 According to the systematic review by Kawasaki et al. 28 that included studies with pregnant women who had T1DM, fetal exposure to maternal hyperglycemia was also associated with overweight during childhood. This shows that glycemic control during pregnancy can have repercussions not only in the neonatal period, but also throughout childhood. This evidence makes us reflect on the CCM, which promoted better glycemic control during pregnancy, as a method of guidance that can favor greater protection to the health of children.

In our study, it was observed that, in the 3 ^rd^ trimester, the mean of postprandial glycemia in the CCM reached the glycemic goal recommended by ADA (< 140 mg/dl), which was not observed in the TM group. 3 The literature is scarce in studies that demonstrate the effects of glycemic control in the late postpartum period of pregnant women with pregestational diabetes, but it is known that postprandial hyperglycemia induces vascular injury and inflammation, and is related to cardiovascular events that can occur throughout the life of the woman. 29

The most recurrent gestational complication was pre-eclampsia, with no difference being found between the groups. The prevalence of pre-eclampsia observed in the present study was higher than that reported in the same maternity hospital for all the pregnant women ( *n* = 4,464) evaluated in 2011 and 2012, for whom the prevalence was 6.74%. 14 This higher rate was already expected since women with DM are two to six times more likely to present with pre-eclampsia. 30

In a study conducted in Brazil by Chaves et al., 31 similar results to those observed in the present study were described, with pre-eclampsia/eclampsia rates of 20.6% for pregnant women with T1DM and of 14.3% for those with T2DM. In a Danish study, pre-eclampsia was diagnosed in 18% of pregnant women with T1DM. 32 The increased chance of pregnant women with DM developing pre-eclampsia and eclampsia can be explained in part by the strong association between insulin resistance and arterial hypertension. The hypothesis currently accepted to explain this association is the action of insulin resistance on the endothelium, hindering the vasodilatory action of nitric oxide and/or facilitating vasoconstriction. 33 An epidemiological study with Brazilian women suggests an association between pre-eclampsia and cardiovascular events subsequent to gestation. 34

Regarding the type of delivery, we found that cesarean section was associated with the presence of gestational hypertensive syndrome. Shen et al. 35 also identified in their study with 7633 Canadian pregnant women an association of the presence of pre-eclampsia with the induction of cesarean delivery (OR = 2.21; 95%CI: 1.66–2.95).

The limitations of the present study are its nonrandomized design and the small size of the convenience sample. Some data was lost due to the characteristics of the study, with some data being collected retrospectively. However, it is noteworthy that the estimated sample size was reached in the study. As the control group was historical, capillary glycemia values were not available. Glycated hemoglobin levels were not analyzed as there were few data available for both groups. Another limitation is the inclusion in the study of pregnant women with different types of DM and, due to the small sample size, the impossibility of analyzing the data across different DM strata. Despite these limitations, similarities were observed between the groups, favoring the interpretation of the findings in relation to the impact of the intervention. It is important to note that this is an unpublished study of pregnant women with DM.

It can be concluded that the CCM nutritional guidance method presented a similar performance to the TM in relation to the outcomes of pregnancy tested in women with pregestational DM.

In the clinical practice, the nutritional orientation method may be a decision shared between the pregnant woman and the nutritionist/doctor. The importance of having a nutritionist in the team of professionals providing antenatal care for women with DM was evidenced in the present study, since it positively affected the outcomes analyzed.
